# Dietary Practices, Nutrient Adequacy, and Nutrition Status among Adolescents in Boarding High Schools in the Kilimanjaro Region, Tanzania

**DOI:** 10.1155/2020/3592813

**Published:** 2020-06-29

**Authors:** Calista Nicholaus, Haikael D. Martin, Neema Kassim, Athanasia O. Matemu, Judith Kimiywe

**Affiliations:** ^1^Department of Food Biotechnology and Nutrition Sciences, Nelson Mandela African Institution of Science and Technology, P.O. Box 447, Arusha, Tanzania; ^2^Department of Food, Nutrition and Dietetics, Kenyatta University, P.O. Box 43844-00100, Nairobi, Kenya

## Abstract

A cross-sectional study was conducted to assess dietary practices, nutrient adequacy, and nutrition status among 164 adolescents aged between 16 and 19 years in boarding secondary schools in the Kilimanjaro region. In-depth interviews and a survey guided by a semistructured and structured questionnaire, including 24-hour recall and food frequency questionnaire techniques, were used to collect information. Nutrition status was assessed using anthropometric measurements and hemoglobin levels. WHO AnthroPlus software and NutriSurvey software were used to analyze anthropometry and dietary data, respectively. Diet in boarding schools was monotonous comprising mainly of cereal-legume meal with low intake of animal sources, fruits, and vegetables. Mean intake of energy, vitamin C, iron, calcium, and zinc was 1392 kcal, 24.8 mg, 9.2 mg, 134.5 mg, and 4.3 mg, respectively, which were below the Recommended Daily Allowance. The average carbohydrate, fat, and protein intake of 471.9 g, 73.7 g, and 80.7 g, respectively, were slightly higher than the Recommended Daily Allowance in both sexes. Male had a significantly higher intake of protein and carbohydrates (*P* < 0.001). Female had a significantly (*P* < 0.001) high intake of fat compared to male adolescents. Overall, 23.1% of the adolescents were anaemic, 25% were overweight, and 6.1% were obese. Boarding secondary schools' diet is monotonous and are inadequate in key micronutrients, iron, zinc, calcium, and vitamin C. There is a coexistence of undernutrition and overnutrition among adolescents in boarding schools. Therefore, monitoring adolescents' dietary intake and nutrition status is a key in preventing adolescents' malnutrition in the short term and diet-related diseases in the long term.

## 1. Introduction

Malnutrition is still prevalent globally with the coexistence of both overnutrition and undernutrition in developing countries affecting people of all ages [1–3]. Adolescents are particularly at risk of malnutrition due to rapid growth and development and changes in dietary habits that may have influenced their nutrient intake [4]. Globally, over 340 million children and adolescents aged 5–19 years are overweight and obese [5]. Overweight and obesity are the main contributors to noncommunicable diseases which account for almost two-thirds (63%) of adult deaths globally [6]. Likewise, the prevalence of underweight (thinness) among children and adolescents is 8.4% for girls and 12.4% in boys [4]. Additionally, micronutrient malnutrition contributes substantially to health risks globally [7]. The most prevalent micronutrient deficiency among adolescents worldwide is iron [8].

In Tanzania, adolescents are affected by malnutrition though this is underreported; little has been done to improve health and nutritional status of adolescents; hence, few data are available at national level which considers the nutrition status of adolescent girls (women of reproductive age which includes women from 15 years of age) only [9–13]. Research shows that about 14.8 % adolescent girls aged between 15 and 19 years were underweight and 12.5% were overweight and obese, and the highest overweight/obesity prevalence was in the Kilimanjaro region [14]. Furthermore, few studies that have focused on the nutritional status of adolescents have revealed the existence of all forms of malnutrition in adolescents [12, 15–20].

The vulnerability of adolescents to malnutrition has been increasing due to inadequate attention in most of the health programs and nutrition interventions. Most nutrition interventions have focused on underfive children and women of reproductive age, particularly pregnant and lactating women leaving out the adolescent group, who are also vulnerable to malnutrition due to the additional nutrient demand for physical growth and development.

Adolescents in boarding schools are fed mainly cereal-legume meals [21, 22]. Cereal-based meals are well documented to contain low contents in key nutrients such as iron, zinc, and calcium [23]. Lack of dietary diversity and the presence of antinutritional factors such as phytates have increased the susceptibility of adolescents to undernutrition [24]. Phytates have inhibitory effects on the bioavailability of these nutrients which are essential in growth and development during adolescence [4].

Dietary and other lifestyle behaviors formed during adolescence contribute to adulthood behaviors [25]. Therefore, school is a good platform for addressing the health and nutrition needs of adolescents as an important step towards reinforcing health behavior in the future [26]. This study therefore aimed at assessing the dietary practices and nutritional status of adolescents in boarding secondary schools.

## 2. Materials and Methods

### 2.1. Study Area, Design, and Sampling Technique

A cross-sectional study was carried out in the Kilimanjaro region, which is located in the northeastern part of Tanzania. The region has wide varieties of foods produced such as bananas, maize, beans, rice, potatoes, and sorghum that might have influenced dietary diversification compared to other regions [27]. It has sixty boarding high schools both private and public, which is the highest number compared to other regions. Out of the sixty schools, fifty-four are located in rural/semirural areas (Table 1) while six are in urban areas. The study involved a total of 31 boarding high schools form six districts, namely, Hai, Siha, Moshi District Council, Same, Mwanga, and Rombo. The selection composed of both private and government-owned schools.

**Figure 1:**
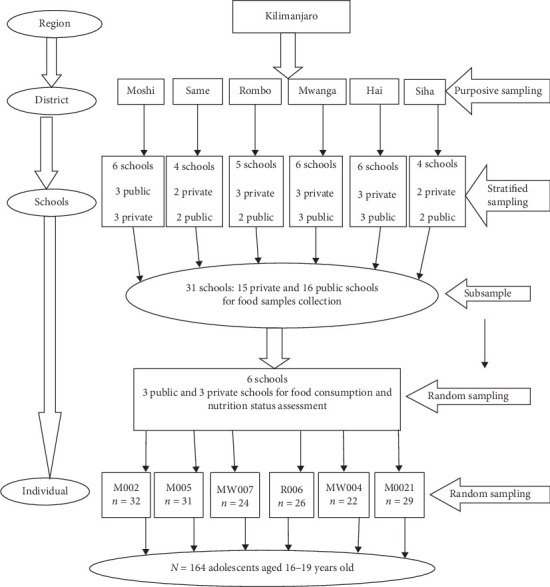
Schematic presentation of the sampling procedure.

A multistage random sampling technique was used, whereby schools in each district were clustered into private or government [28]. A list of boarding high schools in each district was obtained from the respective district office. In each district, at least two and at most three schools were randomly selected from each cluster; hence, 16 public and 15 private schools were recruited to make a total of 31 schools for food sampling (Figure 1) (data not used in this study).

Six schools were subsampled for assessments of food consumption and nutrition status. These schools were randomly selected from the two clusters of private and public schools, and hence, three schools from each cluster were involved making a total of six schools. The sample size was calculated using Fischer's formula [29] considering the prevalence of overweight (12.7%) among adolescents in secondary schools [30]. A non response rate of 10% was considered, and the calculated sample size was 189 adolescents. Of the 189 randomly selected adolescents, 164 adolescents aged between 16 and 19 years (112 females and 52 males) participated in the study (Figure 1). Participating schools and subjects were given special codes to maintain anonymous, and confidentiality of the information was assuared.

### 2.2. Inclusion and Exclusion Criteria

Study participants included boarding high school adolescent students aged 16–19 years who consented to participate in the study and whose school administrators signed consent forms. Adolescents who did not consent and those who were sick were excluded from the study.

### 2.3. Data Collection

In-depth interviews and surveys guided with structured and semistructured questionnaires were used to collect participants' demographic information, dietary information, and anthropometry measurements. The questionnaire consisted of both open- and closed-ended questions. The questionnaires were designed in the English language, translated to Swahili to make it easier for the participants to understand. The questionnaires were pretested among ten students in one boarding school which were not part of this study. Necessary modifications were made before administering the same to the study participants.

### 2.4. Assessment of Nutrition Status

#### 2.4.1. Anthropometry

Anthropometric measurements of weight, height, and age were taken by the researcher and a trained research assistant. Weight was taken with bare feet and light clothing and participants standing upright in the self-calibrating digital weighing scale. Height measurements were taken by using a stadiometer (ADEL MS-120 Medical Scale) measured to the nearest 0.1 cm with the participants standing upright with bare feet. All measurements were taken in a designated room to minimize interference. Anthropometric measurements were used to calculate the BMI and BMI for age which was computed by WHO AnthroPlus software (version v1.0.4). The body mass index (weight (kg)/height (m^2^)) is a reflection of the height and weight of an individual. It is a simple method to determine nutrition status based on weight, height age, and sex [31]. WHO [32] criteria were used to classify nutrition status. The BMI for age *Z*-score categories was used for adolescents aged below 19 years such as <−2 SD (underweight), ≥−2 SD and ≤+1 SD (normal), >+1 SD and ≤+2 SD (overweight), and >+2 SD (obese) while the BMI was used to classify nutrition status of adolescents aged above 19 years such as <18.5 (underweight), 18.5 to 24.9 (normal), 25.0 to 29.9 (overweight), and ≥30 (obese), [33]. Stunting or short stature was determined by height for age *Z*-score less than −2 standard deviation [33].

#### 2.4.2. Hemoglobin Levels

Anaemia is one of the most serious and common nutritional deficiency disorders of public health concern in developing countries [34]. It is the condition of low levels of hemoglobin in the blood [35, 36]. Iron is the main component of hemoglobin, and iron deficiency is estimated to be responsible for over half of all anaemia globally [35]. The hemoglobin level was determined on-site by using a portable battery-operated HemoCue Hb 201^+^ system (HemoCue AB, Angelholm, Sweden) which was tested before use. Hemoglobin level determination was done by a trained laboratory technician. A drop of capillary blood from a finger prick was drawn and filled in a HemoCue cuvette, and results were read within 10 minutes. WHO [37] hemoglobin cutoff points for adolescents were used to assess anaemia as indicated in Table 2.

### 2.5. Dietary Intake

Dietary data were collected using the food frequency questionnaire (FFQ) and 24-hour dietary recall methods. A semiquantitative food frequency questionnaire was used to assess the feeding frequency and habitual diet of adolescents. The method was chosen because it is simple and inexpensive to apply in low-resource settings [38]. A modified FFQ comprised a list of 50 food items confined in twelve food groups, namely, cereals and cereal products, roots, tubers and plantains, legumes, meat and meat products, oils and fat, milk and milk products, fruits, vegetables, beverages, snacks, and others, was used [39]. Each participant was asked to recall how often a certain food and drink was consumed per day, weekly, monthly, rare, and never/not eaten. The questionnaire was adopted from Zack et al. [39] with modification to suit the study population.

Twenty-four-hour dietary recall was used to collect dietary information and the amount of foods and beverages consumed over the past 24 hours inside and outside schools. Participants were asked to recall all foods and drinks taken from when they woke up in the morning up to the time before they went to sleep at night. School meals which were prepared using local recipes, information on the cooking methods, and ingredients used were obtained from teachers responsible for school meals and cooks. The portion size was estimated using cooked food for common food items such as stiff maize porridge, and the estimated portion size was determined using kitchen digital scales. Other common households' utensils were calibrated, and measures were translated into grams equivalent. The household measures, including cups, plates, measuring jars, spoons, customary packing size, and solid foods in pieces or slices, were used to estimate portion size. Also, seasonal fruits were purchased to estimate fruit portion size. Mean daily nutrient intake was calculated by the NutriSurvey for Windows software version 2007 [40].

### 2.6. Ethical Consideration and Logistics

Ethical clearance was sought from the National Health Research Ethics Sub-Committee (NatHREC) of the National Institute for Medical Research (NIMR), Tanzania, with the reference number NIMR/HQ/R.8a/Vol.IX/2730. Permission to conduct this study was also sought from other relevant authorities such as the Regional Administrative Secretary, District Executive Directors, and respective school authorities. The purpose of the study was well explained to the school administrators, students, and caregivers (staff or teacher responsible for meals) before commencement. Assent was sought from students, and their caregivers signed the consent form. The school administrators signed consent forms for their schools and caregivers' consent for the students below 18 years while students aged 18 years and above signed consent form once they agreed to participate in the study. Confidentiality of the information obtained was assured, and participation was voluntary.

### 2.7. Data Analysis

Data were entered in Microsoft Excel and then transferred to IBM Statistical Packages for Social Sciences (SPSS version 23) for coding and analysis. Descriptive statistics such as frequency mean and standard deviation was calculated and presented in tables and graphs. The chi-squared test was used to test differences in nutrition status between boys and girls, private and public schools, and adolescents' nutrition status in different districts. An independent *t*-test was used to test differences in nutrient intake among adolescents. Significant differences were set at *P* value <0.05.

## 3. Results

### 3.1. Sociodemographic Characteristics of the Participants

In total, 164 students from six high schools (three private and three public schools) participated in this study. Out of which, 112 (68.3%) were female and 52 (31.7%) were male. The age of respondents at recruitment ranged between 16 and 19 years with a mean (±SD) of 18.3 (±0.7). About 87.8% of study participants were in the age group of 18 to 19 years and 12.2% were between the age of 16 and 17 years. Participants' mothers, 69 (42.1%), had secondary education, and 79 (48.1%) of participants' mothers were involved in the business as a means of livelihood. Participants' fathers, 45%, had secondary education while 23.2% and 31% had primary and tertiary levels of education, respectively. Likewise, participants' fathers, 65 (39.6%), were formally employed with the rest involved in the business (37.8%) and farming (22.6%). Based on the mode of school ownership, 87 (53.0%) respondents were in private and 77 (47.0%) respondents were in public schools. On the type of school, 79 (48.2%) were in coeducation, 65 (39.6%) were in girls' schools, and 20 (12.2%) were in boys' schools (Table 3).

### 3.2. Schedule of School Meals and Access to Foods outside Schools

School meal frequency per day and access to foods outside the school are presented in Table 4. Participants, 103 (62.8%), said that they ate school meals two to three times per day, and 157 (95.7%) participants reported having a fixed schedule of meals each day. More than one-third, 109 (66.5%), reported having access to foods from outside the school. Snacks, fruits and vegetables which were not part of school meals were accessed from outside the schools by 57(52.3 %) and 14(12.8%), respondents respectively. About 47 (43.1%) of respondents preferred foods from outside due to varieties.

### 3.3. Consumption of Cereals, Roots, Tubers, Bananas, and Legumes

Cereals and cereal products were frequently consumed more than seven times per week; predominantly, stiff maize porridge (*ugali*) was consumed by 68 (41.5%) respondents followed by thin maize porridge (*uji*) by 67 (40.9%) respondents. Other cereal-based foods such as boiled rice were consumed by 106 (64.6%) respondents 3 to 4 times per week, and 53 (32.3%) respondents consumed white bread/bread rolls seven times per week for breakfast. Almost all respondents, 158 (96.3%), consumed kidney beans more than seven times per week (Table 5).

### 3.4. Consumption of Meat, Chicken, Eggs, Fish/Sardines, Milk and Milk Products, and Fats/Oil

Animal-source foods were occasionally consumed as presented in Table 6. Results indicated that almost half of the respondents, 84 (51.2%), consumed meat 1 to 2 times per week. Eggs, fish/sardines, and milk were less consumed by 11 (6.7%), 7 (4.2%), and 8 (4.9%) respondents, respectively. Cooking oils were consumed by all respondents (100%). Groundnuts, margarine, and peanut butter were rarely consumed by few respondents, 12.2%, 8.4%, and 7.9%, respectively.

### 3.5. Consumption of Seasonal Fruits, Leafy Vegetables, Beverages, Snacks, and Other Items

The fruits mostly consumed were ripe bananas, consumed by 59 (36%) respondents, followed by oranges, consumed by 51 (31.1%) respondents 1 to 2 times per week. Ripe bananas were commonly consumed because bananas are readily available in the region. About 9 (5.5%) respondents consumed fruit juice rarely. Leafy vegetables were consumed by 57 (34.7%) respondents 1 to 2 times per week (Table 7).

Black tea (tea without milk) was frequently consumed, and 71 (43.3%) respondents consumed black tea seven times per week as breakfast or as part of a midmorning snack. Also, carbonated drinks (soda) were rarely consumed by 36 (22.0%) respondents. Processed snacks such as biscuits, crisps, and sweets were rarely consumed by 36 (22.0) respondents. Other items such as chili sauce, tomato sauce, and honey were consumed by fewer respondents, 22 (13.4%), 1 to 2 times per week.

### 3.6. Energy and Nutrient Intake

Data from the 24-hour dietary recall indicate that the average energy intake is 1311 kcal for male and 1473 kcal for female which are less than the Recommended Daily Allowances (Table 8). An independent-samples *t*-test was performed to compare the average energy intake between male and female adolescents. The mean intake of protein (80.7 g) and carbohydrates (471.9 g) was slightly above the Recommended Daily Allowance for the adolescents. There was a significant difference in the average intake of protein, carbohydrates, and fat between female and male adolescents (*P* < 0.001). Male adolescents had significant (*P* < 0.001) intake of protein and carbohydrate of 93.9 g and 535.6 g, respectively, as compared to their female counterparts. On the other hand, female adolescents had a significant intake of fat (89.0 g) compared to male (*P* < 0.001).

The average intake of micronutrients, vitamin C (24.8 mg), iron (9.2 mg), calcium (134.5 mg), and zinc (4.3 mg), was below the RDA. Most adolescents did not meet the Recommended Daily Allowance for these micronutrients (Table 8). Iron intake of 75% of adolescents was below the RDA while 70.7% did not meet the RDA for vitamin C. Likewise, 100% and 97% of adolescents did not meet the RDA for calcium and zinc, respectively. There was a significant difference (*P*=0.018) in the mean iron intake between male and female (Table 8). In terms of gender, the majority of female adolescents were below the RDA for micronutrients such as 87.5%, 71.0%, 100%, and 97.3% for iron, vitamin C, calcium, and zinc, respectively (Table 9). The independent-samples *t*-test was used to compare the mean intake among adolescents in age categories from 16 to 17 and 18 to 19 years. Consumption of various nutrients was not significant in the two age categories as indicated in Table 8.

### 3.7. Nutrition Status

**Figure 2:**
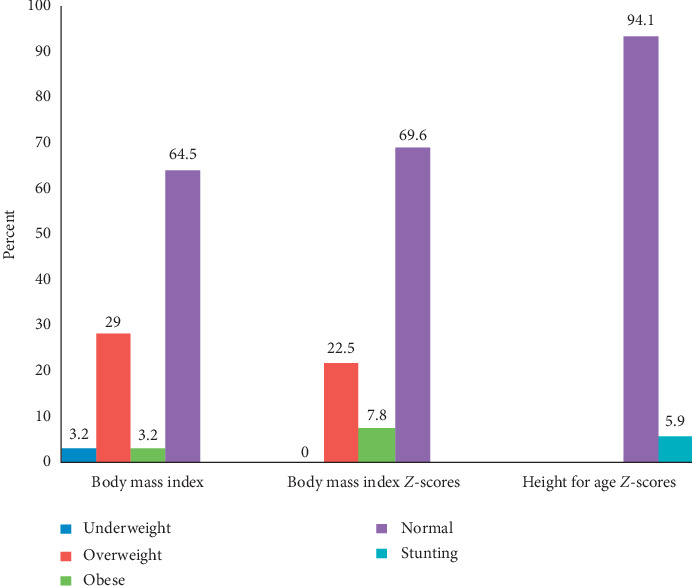
Nutritional status of adolescents. Body mass index, BM1 for age, and height for age *Z*-score cutoffs for adolescent boys and girls [32].

The mean height among adolescents was 160.9 cm. There was a significant difference in height between female and male adolescents (*P* < 0.001). Male adolescents were taller (165.5 cm) compared to female (158 cm). The mean BMI of female adolescents was significantly higher by 23.8 ± (3.8) (*P*=0.022) compared to male 22.5 ± (3.1). Findings on adolescents' BMI indicated that 41 (64.5%) were normal, while 2 (3.2%) were underweight, 18 (29.0%) and 2 (3.2) were overweight and obese, respectively. Moreover, the nutrition status by the BMI for age *Z*-scores showed that 23 (22.5%) were overweight, 8 (7.8%) were obese, and the majority, 71 (69.6%), were normal. In terms of gender, 16 (88.9%) female adolescents were overweight and 5 (62.5%) were obese compared to male. Height for age *Z*-scores indicated that 96 (94.1%) were normal and 6 (5.9%) were stunted (<−2 SD) (Figure 2).

### 3.8. Hemoglobin Levels

The prevalence of anaemia among adolescents was 23.1%. The mean hemoglobin level was 13.6 g/dl. There was a significant difference in mean hemoglobin levels between female and male adolescents (*P* < 0.05). Out of the total adolescent boys and girls (*n* = 164), 17 (15.2%) girls had mild anaemia and 14 (12.5%) had moderate anaemia; in case of boys, 11.5% and 2.0% had mild and moderate anaemia, respectively. There was no severe anaemia found among adolescents in this study (Table 10).

## 4. Discussion

Adolescents' diet in boarding schools in developing countries is confined with traditional starchy staples, mainly cereals accompanied by legumes and little consumption of animal foods, micronutrient-rich fruits, vegetables, and lack of quality dietary diversity [28, 41, 42]. Boarding schools' menus in this study were repetitive with fixed dishes on each day, mainly thin maize porridge (*uji*), stiff maize porridge (*ugali*), beans, maize and bean meal (*kande*), and rice, making it very monotonous and giving adolescents no other food choices, they consume whatever the school provides. Monotonous diets are generally deficient in one or more essential nutrients, hence increasing risks for malnutrition [43]. The quality and quantity of school meals are very essential to meet the nutritional needs of adolescents [44, 45]. The provision of diversified diet in schools enhances not only healthy eating but also food choices which enable adolescents to establish healthier dietary behavior and contribute to the prevention of nutrition-related diseases/conditions later in life [46, 47].

Maize and legumes are the major sources of energy, carbohydrates, and proteins among the population of Tanzania [48, 49]. Maize and legumes are growing in varied agroecological zones in Tanzania, hence are readily available, and can be easily accessed in nearby regions in case of deficit [50, 51]. On the other hand, a diet rich in cereals and legumes is usually inadequate to meet micronutrient requirements [24]. Furthermore, cereals and legumes contain a high amount of phytates which are inhibitors of micronutrient absorption such as iron and zinc [23, 52], and these nutrients are essential for adolescents' growth and development [44].

Findings show that adolescents had energy intake below the RDA. This was consistent with the findings of a study from Kilosa rural adolescents [16], India [53], and Zambia [54] which reported low energy intake among adolescents. However, the adolescents' tendency to underreporting/misreporting food intake might have contributed to the underestimation of energy intake [55].

The intake of proteins and carbohydrates was slightly higher above the RDA. This has been contributed by the high intake of kidney beans and maize-based foods as the main sources of proteins and carbohydrates in boarding schools. Male participants had a significant high intake of proteins and carbohydrates as compared to their female counterparts (*P* < 0.001). The difference is more related to the portion size consumed between male and female adolescents. Even so, the consumption of proteins was observed to be higher but the protein sources were mainly from plant sources which jeopardize the interpretation of these findings in terms of quantity of proteins consumed. Animal-source proteins such as meat were consumed at a very low rate in boarding schools. Others such as fish, eggs, and milk were excluded from most of the schools' menu. Animal-source proteins have been classified as good quality proteins due to the presence of essential amino acids that are easily digestible and bioavailable in the body [56]. Animal proteins are a good source of essential micronutrients [57]. However, animal-source proteins are considered expensive in terms of costs; hence, most of the boarding schools do not afford its regular consumption and others have omitted them completely from the school menu.

Fat intake among adolescents in this study was slightly higher above the RDA. These findings were inconsistent with findings from India, Nigeria, and Kilosa-Tanzania which reported low-dietary-fat intake among adolescents [16, 42, 58]. In line with this research, studies from Spain, South Africa, Benin, and Nigeria reported high fat intake among adolescents [59–63]). Adequate intake of fat acts as a source of energy enhances the absorption of fat-soluble vitamins, and it is an essential component of the cell membrane and certain hormones [64]. Moreover, fat improves the palatability of foods [65]. The WHO recommended that less than 30% of the daily energy must be from fat [66]. On the other hand, a high intake of fat during adolescence is associated with increased risks of overweight/obesity, hence increasing risks of nutrition-related diseases later in life [67]. Findings from the study found that cooking oils added to school meals and bread spreads which students purchase on their own were the main sources of fat. Significantly, female had higher fat intake than male which was contributed by the consumption of high-fat foods such as margarine and peanut butter. Surprisingly, female adolescents regularly enhanced the flavor of their meals by adding peanut butter and margarine, particularly in thin maize porridge *(uji*) and maize and bean meal *(kande*).

Micronutrient adequacy during adolescence is essential not only to support adolescents' health status but also to the future health in adulthood [68, 69]. The increased demand for micronutrients during adolescence is contributed by a growth spurt and hormonal changes [44]. Micronutrient deficiencies during adolescents can cause impaired growth, delayed sexual maturation, and poor reproductive outcomes later in life, particularly among girls [70]. Findings in this study indicated that micronutrient intake such as calcium, zinc, iron, and vitamin C was below the RDA for both sexes. Generally, an inadequate intake of calcium, iron, and zinc was contributed to a low intake of animal-source foods. In this study, none of the study population had sufficient intake of calcium. Adequate calcium intake is important for bone development during adolescence and for the prevention of osteoporosis in adulthood [44, 71]. About 45% of adult bone mass development occurred during adolescence [70].

With regards to iron intake, findings indicate that the majority of adolescents were below the RDA. Consistent to these findings, reports from India and Ethiopia indicated that school-going adolescents had low iron intake due to high consumption of non-hem rich foods and foods that inhibit the absorption of iron [58, 72]. Findings indicated that adolescents in private schools had low iron intake compared to their counterparts. It was expected that adolescents from private schools could have a better iron intake as compared to public schools because private school adolescents are from affluent families and are expected to have a better diet from their schools, which was not the case in this study. Even though the economic status of the individual adolescent was not assessed, the differences in fees between private and public schools could tell the differences. Moreover, adolescents in most of the private boarding schools are bound by strict school rules that prohibit them from accessing varieties of foods outside school compound which might have helped to enhance the diversity of their school cereal-legume meal. Adequate iron during adolescence is important for building iron stores and maintenance of hemoglobin concentration, cognitive ability, and muscle cells [44, 68]. Inadequate intake of iron may lead to anaemia which may affect adolescents' growth and development [73].

Vitamin C is an antioxidant that has a preventive role of noncommunicable diseases such as some cancers and cardiovascular diseases [74]. Also, vitamin C is an important enhancer in the absorption of nonhem iron in the body [74–76]. The majority of adolescents in the study population were below the RDA for vitamin C. Sources of vitamin C for boarding scholars were limited, and fresh oranges were observed to be the main source. However, oranges were rarely consumed due to seasonality and availability plus the fact that one has to purchase, and in some schools, it is not part of school meals even during the high season. Other sources such as green leafy vegetables are, though, rarely consumed, but most of the time they are overcooked as they mixed with beans; hence, vitamin C might be lost during cooking. Vitamin C is water-soluble and easily degraded by high temperatures [77].

The findings of this study revealed the coexistence of undernutrition and overnutrition among boarding school adolescents. The prevalence of 25.0% overweight and 6.1% obesity among boarding school adolescents found in this study is relatively higher than 12.7% reported in school adolescents in Babati [19] and Bahrain and Ethiopia [72, 78, 79]. Findings show that the prevalence of overweight (30%) in female adolescents was higher than male adolescents (13.4%). Similarly, findings from a study in Kenya reported 18.7% overweight among secondary school girls [80]. A study in Ethiopia reported overweight in school adolescents of 5.3% male and 16.2% female [28]. Adolescent girls overweight/obesity are attributable to hormonal changes that influence the accumulation of fat mass in adolescent girls than boys [81]. Moreover, male adolescents are more physically active compared to female ones [82], and sometimes, boys in boarding schools are engaged in vigorous activities to include various sports which make them more active. Also, there is an existing perception among the African community that fatness with round body shapes among females is associated with beauty; hence, female adolescents do not feel vulnerable due to overweight/obesity [83].

The higher prevalence of overweight and obese among adolescents in this study is alarming; it signifies the existence of nutrition transition from undernutrition to overnutrition. In comparison to previous studies which were done in primary schools in Dar-es-salaam, there is an increase in the prevalence of overweight and obesity among adolescents in Tanzania. Muhihi et al. [84] reported 9.8% overweight and 5.2% obese and Pangani [17] reported 15.9% and 6.7% for overweight and obese, respectively, among primary school-going children [17, 84]. However, at a national level prevalence of overweight and obesity among adolescents, girls (15–19 years) stand at 12.5%. The Kilimanjaro region is among the regions with the highest prevalence of overweight/obesity [14].

A sedentary lifestyle among boarding school adolescents could be a reason for the increased prevalence of overweight/obesity among boarding scholars. Though the level of physical activities was not examined, the school academic timetable is convincing to justify this. Due to the hectic academic schedule, these adolescents might have used most of their time in the classroom and less in physical activities. Also, adolescents in boarding schools have restricted movement compared to adolescents in day schools who sometimes walk a long distance before reaching schools. A study which was done in Kinondoni revealed that the risk of being overweight/obese was lower in school children who walk to and from schools [85]. Berbada et al. [86] found that the risk of adolescents with a sedentary lifestyle to being overweight and obesity is high. On the other hand, Ogechi [42] revealed that adolescents in boarding schools spend less energy and are involved in a sedentary lifestyle. Moreover, the dietary practices of adolescents might have influenced the increased prevalence of overweight and obesity. High intake of carbohydrates and less consumption of fruits and vegetables among adolescents in boarding schools might have contributed to the increased prevalence of overweight and obesity. Some studies reported an association between low fruits and vegetable consumption and overweight and obesity among adolescents [28, 87, 88]. Obesity has been identified as a risk factor attributed to various non communicable diseases including type 2 diabetes, cardiovascular diseases, hypertension, and certain cancers [88]. The onset of obesity and overweight during adolescence may persist to adulthood and hence pause greater health effects in the future [89]. Therefore, the findings of this study provide insight into the need for routine assessment and nutrition intervention among boarding school adolescents.

On the other hand, the prevalence of underweight (1.2%) and stunting (3.7%) reported in this study was low compared to that reported in Ethiopia among school-going adolescents with underweight (13.4%) and stunting (3.6%) [72] and Nigeria with underweight (6.4%) and stunting (1.8%) [31]. Adolescent stunting is a reflection of chronic malnutrition which resulted from prolonged poor nutrition, infection, and environmental stress accumulated from fetal through adolescence [4].

Micronutrient deficiency, particularly anaemia, was prevalent among boarding school adolescents. From the study, the overall prevalence of anaemia among adolescents was 23.1%. Female adolescents had a higher prevalence of 18.9% compared to 4.3% male. This was consistent with findings reported in Tanzanian suburban areas which indicated adolescent anaemia of 14.5% and 7.9% for female and male, respectively [90]. The prevalence in this study is lower than that reported in India (62% females and 46.0% males were anaemic) [69] and Saudia Arabia (34.2% females and 16.7% males were anaemic) [91]. Anaemia causes are multifactorial but mainly is due to iron deficiency [20, 90]. It is well stipulated that 50% of anaemia cases are caused by iron deficiency [92, 93]. Iron intake in adolescents may be poor due to inadequate intake as a result of changes in dietary habits and insufficient iron in the diet due to poor bioavailability [94]. Moreover, the prevalence of anaemia in adolescents could be also due to physiological changes in both female and male adolescents [44]. The low prevalence of anaemia in male adolescents could be attributed to the increase in hemoglobin concentration contributed by sexual maturation and reduced requirements that occurred after the growth spurt [91]. Likewise, the higher prevalence of anaemia in the female is due to rise in iron requirements due to menstruation [44, 95]. Anaemia among female adolescents has consequences on their growth and development, the cognitive ability, and hence affects academic performances and irregular menstrual cycle [96]. It also affects physical fitness and work productivity and may lead to reproductive complications [94]. Therefore, anaemia in female adolescents, if not timely managed, may be a source complication in the entire female life cycle and hence intergenerational malnutrition [91].

### 4.1. Strength and Limitations

The strength of this study is that it was conducted among boarding scholars in rural and semirural settings of the Kilimanjaro region. The study was able to determine the dietary practices and nutrition status of adolescents in boarding schools.

However, the interpretation of these findings must be done with caution due to some limitations such as being a cross-sectional study was unable to show causal and effect relationship. Findings of this study are limited to few schools in one region, therefore may not be generalized to all adolescents in the whole country. A broader study is recommended.

Other factors that contribute to overweight/obesity such as physical activities, hormonal changes during puberty, and genetics were not assessed. Moreover, other factors that may contribute to anaemia apart from diet, factors such as menstruation, infectious diseases, and parasites such as helminthes, were not assessed in this study. The study did not assess the association of anaemia with nutrient intake using biomarkers.

## 5. Conclusion and Recommendations

Diet of adolescents in boarding secondary schools is monotonous comprising mainly of cereals and legumes with minimal animal sources, fruits, and vegetables which are important in the provision of key micronutrients. Moreover, boarding school diet is inadequate in essential micronutrients of iron, zinc, calcium, and vitamin C based on adolescents' Recommended Daily Allowance according to age and sex. The coexistence of overnutrition and undernutrition in boarding schools found in this study is an alert showing the importance of routine nutrition assessment among adolescents. This will in turn help to reduce the burden of some noncommunicable diseases in Tanzania. School administrators should put emphasis on dietary diversity in the school menu with inclusion of nutrient-rich foods such as animal sources, fruits, and vegetables to address malnutrition among boarding school adolescents. Monitoring of the adolescents nutrition and health status is crucial, and the health sector should take the lead in routine health and nutrition assessment, monitor level of physical activities, dietary practices, and nutrition status among boarding scholars. A broader study to assess micronutrient deficiency using biomarkers is recommended.

## Figures and Tables

**Table 1:** Boarding high schools in rural and semirural areas of the Kilimanjaro region.

District	Public	Private	Total
Rombo	2	3	5
Hai	3	6	9
Siha	4	3	7
Moshi (R)	6	11	17
Same	2	5	7
Mwanga	5	4	9
Total	22	32	54

**Table 2:** Hemoglobin levels to define adolescents' anaemia.

Category	Hemoglobin cutoff points in g/dL	
	Girls	Boys
Nonanaemia	12 or above	13 or above
Mild anaemia	11.0–11.9	11.0–12.9
Moderate anaemia	8–10.9	8–10.9
Severe anaemia	Below 8	Below 8

Refer to [37].

**Table 3:** Sociodemographic characteristics of participants (*n* = 164).

Characteristics	*n*	%

Mean (standard deviation)	18.3 (0.7)	
Age (years)		
16-17	20	12.2
18-19	144	87.8
Gender		
Female	112	68.3
Male	52	31.7
The education level of the mothers		
Primary education	47	28.6
Secondary education	69	42.1
Tertiary education	48	29.3
The education level of the fathers		
Primary education	38	23.2
Secondary education	75	45.7
Tertiary education	51	31
Occupation of the mother		
Employed	48	29.3
Farmer	37	22.6
Businesswoman	79	48.1
District		
Rombo	26	15.9
Moshi	94	57.3
Mwanga	44	26.8
School ownership		
Private	87	53.0
Public	77	47.0
School type		
Girls only	65	39.6
Boys only	20	12.2
Coeducation	79	48.2

**Table 4:** School meals schedule and access to foods outside the school.

Variable	Attributes	*n*	%
Frequency of eating school meals	2-3 times per day	103	62.8
	4 times per day	61	37.2

Schedule of meals on each day	Yes	157	95.7
	No	7	4.3

Access to foods out of school	Yes	109	66.5
	No	55	33.5

Types of foods accessed outside	Snacks	57	52.3
	Main meals	38	34.9
	Fruits and vegetables	14	12.8

Reasons for accessing foods from outside	Varieties	47	43.1
	Nutrition	17	15.6
	Good taste	23	21.1
	Availability	22	20.2

**Table 5:** Consumption of cereals, roots, tubers, bananas, and legumes.

Food group	Frequency of consumption						
	1-2 times/wk	3-4 times/wk	5-6 times/wk	7≥ times/wk	1-2 times/month	Rare	Never
	*n* (%)	*n* (%)	*n* (%)	*n* (%)	*n* (%)	*n* (%)	*n* (%)
Cereals and cereal products							
Thin maize porridge	13 (7.9)	16 (9.8)	27 (16.5)	67 (40.9)	—	—	41 (25)
Stiff maize porridge (*ugali*)	7 (4.3)	66 (40.2)	17 (10.3)	68 (41.5)	—	—	6 (3.7)
Maize and bean meal (*kande)*	70 (42.7)	65 (39.7)	10 (6.1)	15 (9.1)	—	—	4 (2.4)
Boiled rice	34 (20.7)	106 (64.6)	24 (14.6)	—	—	—	—
Meat pilau	29 (17.0)	—	—	—	18 (10.9)	11 (6.7)	106 (64.6)
White bread/bread rolls	17 (5.5)	27 (16.5)	38 (33.2)	53 (32.3)	—	5 (3.0)	24 (14.6)
Buns	22 (13.4)	28 (17.1)	14 (8.5)	19 (11.6)	6 (3.7)	—	82 (50)
Chapati	7 (4.2)	5 (3.0)	2 (1.2)	18 (11.0)	—	—	132 (80.5)
Roots, tubers, and plantains							
Roasted potatoes (*kachori)*	10 (6.1)	4 (2.4)	—	—	—	—	150 (91.5)
Banana stew	—	—	—	—	3 (1.8)	13 (7.9)	148 (90.2)
Legumes							
Kidney beans	—	3 (1.8)	3 (1.8)	158 (96.3)	—	—	—

— represents a particular food item was not consumed.

**Table 6:** Consumption of meat, chicken, eggs, fish/sardines, milk and milk products, and fats/oil.

Food group	Frequency of consumption						
	1-2 times/wk	3-4 times/wk	5-6 times/wk	7≥ times/wk	1-2 times/month	Rare	Never consumed
	*n* (%)	*n* (%)	*n* (%)	*n* (%)	*n* (%)	*n* (%)	*n* (%)
Sardines							
Meat chicken, fish/sardines, and eggs							
Meat (beef)	84 (51.2)	—	—	—	36 (22.0)	23 (14)	21 (12.8)
Eggs	11 (6.7)				3 (1.8)	30 (18.3)	120 (73.2)
Fish/sardines	7 (4.2)	5 (3.0)	—	—	—	—	152 (92.7)
Milk and milk product							
Cow's milk	5 (3.0)	8 (4.9)	—	—	—	—	151 (92.1)
Fats and oil							
Cooking oil (sunflower, OKI Korie, Safi)	—	—	—	164 (100)	—	—	—
Groundnuts	15 (8.1)	4 (2.4)	—	3 (1.8)	2 (1.2)	20 (12.2)	120 (73.2)
Margarine	8 (4.8)	4 (2.4)	7 (4.3)		6 (3.7)		137 (83.5)
Peanut butter	10 (6.0)	10 (6.1)	5 (7.9)	4 (2.4)	—	13 (7.9)	122 (74.4)

— represents a particular food item was not consumed.

**Table 7:** Consumption of seasonal fruits, leafy vegetable, beverages, snacks, and other items.

Food group	Frequency of consumption						
	1-2 times/wk	3-4 times/wk	5-6 times/wk	7≥ times/wk	1-2 times/month	Rare	Never consumed
	*n* (%)	*n* (%)	*n* (%)	*n* (%)	*n* (%)	*n* (%)	*n* (%)
Seasonal fruits							
Ripe bananas	59 (36)	22 (13.4)	2 (1.2)	11 (6.7)	2 (1.2)	50 (30.5)	18 (11.0)
Avocado	17 (10.3)	20 (12.1)	3 (1.8)	14 (8.5)	—	26 (15.8)	84 (51.2)
Oranges	51 (31.1)	9 (5.5)	—	—	3 (1.8)	21 (12.8)	87 (53.0)
Fruit juice	7 (4.2)	4 (2.4)	—	—	—	9 (5.5)	144 (87.8)
Vegetables							
Leafy vegetables	57 (34.7)	8 (4.2)	—	6 (3.7)	—	48 (29.3)	45 (27.4)
Beverages							
Black tea	8 (4.9)	30 (18.2)	41 (25)	71 (43.3)	—	—	14 (8.5)
Milk tea	6 (3.6)	2 (1.2)		2 (1.2)			154 (93.9)
Carbonated drinks (soda)	21 (12.8)	15 (9.1)	8 (4.9)	—	22 (13.4)	36 (22)	62 (37.8)
Processed snacks (biscuits, crisps, sweets)	30 (18.3)	21 (12.8)	3 (1.8)	8 (4.9)	—	36 (22.0)	66 (40.2)
Other items (honey, chili sauce, tomato sauces)	5 (3.0)	9 (5.5)	7 (4.3)	22 (13.4)	—	9 (5.5)	119 (72.6)

— represents a particular food item was not consumed.

**Table 8:** Energy and nutrient intake of the foods consumed by adolescents in boarding secondary schools.

Nutrients	RDA	Male, M (SD) *n* = 52	Female, M (SD) *n* = 112	*t*-test	*P* value
Energy (kcal)	2162.70	1311.7 (442.4)	1473.8 (567.7)	−1.989	0.049
Protein (g)	63.90	93.9 (28.4)	67.6 (20.9)	5.986	<0.001^*∗*^
Fat (g)	73.40	58.3 (32.3)	89.0 (38.3)	−5.013	<0.001^*∗*^
Carbohydrates (g)	308.70	535.6 (177.2)	408.3 (117.6)	4.719	<0.001^*∗*^
Iron (mg)	9.00 and 12.50	10.6 (7.7)	7.8 (4.9)	2.430	0.018^*∗*^
Vitamin C (mg)	30.00	26.7 (53.8)	22.9 (23.9)	0.485	0.630
Calcium (mg)	450.00	139.2 (97.3)	130.8 (81.6)	0.576	0.565
Zinc (mg)	6.5, 9.4, and 10.20	4.6 (2.9)	4.0 (2.0)	1.324	0.190

Age distribution					
Nutrients	RDA	16-17 M (SD) *n* = 20	18-19 M (SD) *n* = 144	*t*-test	*P* value
Energy (kcal)	2162.70	1482.2 (544.9)	1414.1 (535.2)	0.533	0.595
Protein (g)	63.90	69.4 (19.4)	76.8 (27.2)	−1.185	0.238
Fat (g)	73.40	95.2 (39.6)	77.1 (38.7)	1.955	0.052
Carbohydrates (g)	308.70	415.2 (123.2)	453.3 (154.1)	−1.058	0.292
Iron (mg)	9.00 and 12.50	6.6 (4.5)	8.9 (6.2)	−1.642	0.103
Vitamin C (mg)	30.00	18.8 (21.7)	24.8 (37.5)	−0.705	0.482
Calcium (mg)	450.00	135.6 (117.2)	133.1 (82.0)	0.119	0.906
Zinc (mg)	6.5, 9.4, and 10.20	3.6 (1.9)	4.3 (2.4)	−1.327	0.186

^*∗*^Statistically significant (*P* < 0.001); nutrient intake was computed by NutriSurvey for Windows 2007 and the Tanzania Food Composition Table, 2008. RDA represents the Recommended Daily Allowances, ≥RDA represents above or equal to the RDA, and <RDA represents below the RDA.

**Table 9:** Schools' ownership and gender vs. energy and nutrient intake.

Nutrients	School ownership		*P* value	Gender		*P* value
	Private *n* (%)	Government *n* (%)		Male *n* (%)	Female *n* (%)	
Energy			1.004 (0.316)			5.473 (0.019)
<RDA	78 (89.7)	65 (84.4)		50 (96.2)	93 (83.0)	
≥RDA	9 (10.3)	12 (15.6)		2 (3.8)	19 (17.0)	
Protein			0.043 (0.836)			15.505 (0.0001)
<RDA	33 (37.9)	28 (36.4)		8 (15.4)	53 (47.3)	
≥RDA	54 (62.1)	49 (63.6)		44 (84.6)	59 (52.7)	
Fat			3.423 (0.064)			7.131 (0.008)
<RDA	36 (41.4)	43 (55.8)		33 (63.5)	46 (41.1)	
≥RDA	51 (58.6)	34 (44.2)		19 (36.5)	66 (58.9)	
Carbohydrates			1.380 (0.240)			1.775 (0.183)
<RDA	14 (16.1)	18 (23.4)		7 (13.5)	25 (22.3)	
≥RDA	73 (83.9)	59 (76.6)		45 (86.5)	87 (77.7)	
Iron			5.949 (0.015)			29.436 (0.0001)
<RDA	72 (82.8)	51 (66.2)		25 (48.1)	98 (87.5)	
≥RDA	15 (17.2)	26 (33.8)		27 (51.9)	14 (13)	
Vitamin C			0.718 (0.397)			0.007 (0.935)
<RDA	64 (55.2)	52 (44.8)		37 (71.2)	79 (71.0)	
≥RDA	23 (47.9)	25 (52.1)		15 (28.8)	33 (29.5)	
Calcium			—			—
<RDA	87 (53.0)	77 (47.0)		52 (100)	112 (100)	
≥RDA	—	—		—	—	
Zinc			0.353 (0.553)			0.164 (0.686)
<RDA	85 (53.5)	74 (46.5)		50 (96.2)	109 (97.3)	
≥RDA	2 (40.0)	3 (60.0)		2 (3.8)	3 (2.7)	

Significance difference was tested at 95% CI, *P* < 0.005, independent *t*-test. Nutrient intake was computed by NutriSurvey for Windows 2007 and the Tanzania Food Composition Table, 2008. RDA represents the Recommended Daily Allowances, >RDA represents above or equal to the RDA, and <RDA represents below the RDA.

**Table 10:** Distribution of anaemia by gender among adolescents.

Variable	Female *n* (%)	Male *n* (%)
Moderate anaemia	14 (22.5)	1 (2.0)
Mild anaemia	17 (15.2)	6 (11.5)
Nonanaemic	81 (72.3)	45 (86.5)

Hemoglobin classification based on [37].

## Data Availability

The data sets used for this study are available from the corresponding author upon request.
